# A Mechanistic View of the Role of E3 in Sumoylation

**DOI:** 10.1371/journal.pcbi.1000913

**Published:** 2010-08-26

**Authors:** Melda Tozluoğlu, Ezgi Karaca, Ruth Nussinov, Türkan Haliloğlu

**Affiliations:** 1 Polymer Research Center & Chemical Engineering Department, Bogazici University, Istanbul, Turkey; 2 Basic Science Program, SAIC-Frederick, Inc., Center for Cancer Research Nanobiology Program, NCI-Frederick, Frederick, Maryland, United States of America; 3 Sackler Institute of Molecular Medicine, Department of Human Genetics and Molecular Medicine, Sackler School of Medicine, Tel Aviv University, Tel Aviv, Israel; Columbia University, United States of America

## Abstract

Sumoylation, the covalent attachment of SUMO (Small Ubiquitin-Like Modifier) to proteins, differs from other Ubl (Ubiquitin-like) pathways. In sumoylation, E2 ligase Ubc9 can function without E3 enzymes, albeit with lower reaction efficiency. Here, we study the mechanism through which E3 ligase RanBP2 triggers target recognition and catalysis by E2 Ubc9. Two mechanisms were proposed for sumoylation. While in both the first step involves Ubc9 conjugation to SUMO, the subsequent sequence of events differs: in the first E2-SUMO forms a complex with the target and E3, followed by SUMO transfer to the target. In the second, Ubc9-SUMO binds to the target and facilitates SUMO transfer without E3. Using dynamic correlations obtained from explicit solvent molecular dynamic simulations we illustrate the key roles played by allostery in both mechanisms. Pre-existence of conformational states explains the experimental observations that sumoylation can occur without E3, even though at a reduced rate. Furthermore, we propose a mechanism for enhancement of sumoylation by E3. Analysis of the conformational ensembles of the complex of E2 conjugated to SUMO illustrates that the E2 enzyme is already largely *pre-organized* for target binding and catalysis; E3 binding shifts the equilibrium and enhances these pre-existing populations. We further observe that E3 binding regulates allosterically the key residues in E2, Ubc9 Asp100/Lys101 E2, for the target recognition.

## Author Summary

Post-translational modifications constitute key regulatory mechanisms in the cell. One of these modifications is the tagging of the target protein with a smaller molecule. SUMO is such a ubiquitin-like tag protein, and sumoylation is the process of tagging proteins with SUMO. The malfunctioning of sumoylation is linked with diseases such as Alzheimer's, Parkinson's, and cancer. Based on experimental observations, two paths were suggested for sumoylation, the first and more efficient involves the E1, E2 and E3 enzymes; the second only the E1 and E2. Here we investigate these alternative paths of sumoylation. Our results offer an explanation for how sumoylation can take place with only the E1 and E2 enzymes, and for the mechanistic role of E3. They emphasize that E2 bound to SUMO is already pre-organized for the transfer of SUMO to a target protein and E3 binding further stabilizes the conformations, shifting the ensemble and thus increasing the efficiency of the sumoylation.

## Introduction

Protein function is regulated by numerous mechanisms, one of which is post-translational modification. Covalent binding of ubiquitin (Ub) and ubiquitin-like (Ubl) modifiers to target proteins constitute a key step in cellular processes including differentiation, apoptosis, cell cycle, and stress response [1]–[4]. Here, we focus on one member of the Ubl super-family, SUMO, with the aim of figuring out the mechanism through which SUMO is conjugated to its target proteins.

**Figure 1:**
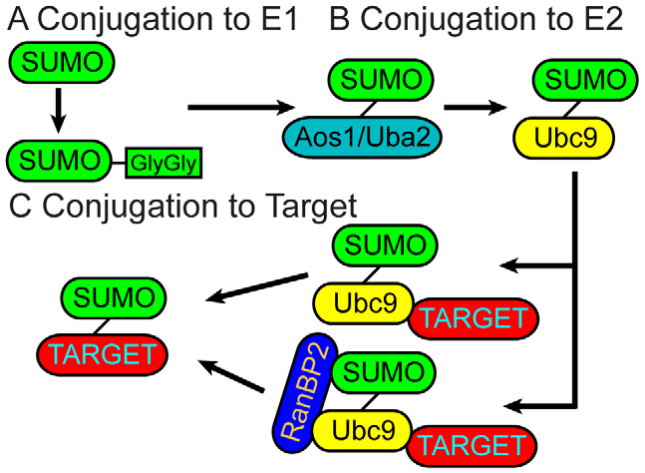
Sumoylation mechanism by E1-E2 and E1-E2-E3 enzymes. (A) Produced as an inactive precursor, the SUMO protein is cleaved, exposing its Gly-Gly motif, and gets activated. (B) Active SUMO is transferred by E1 enzyme Aous1/Uba2 heterodimer to the E2 enzyme Ubc9. (C) Two alternative pathways can follow as the third step. Ubc9 can directly transfer SUMO to specific targets (top). Alternatively RanBP2, an E3 enzyme, can also join the complex, increasing the Ubc9 catalytic activity and transferring SUMO to its targets (bottom).

SUMO-1 (Small ubiquitin-like modifier, also known as PIC1, UBL1, GMP1, Sentrin), -2, -3 and -4 exist in mammals [5]–[10]. Sumoylation can change the proteins' intracellular localization, interaction patterns with other proteins and modifications by other post-translational events. It is important in development [11] and is related to cancer drug resistance [12], [13]. For simplicity, below, SUMO refers to SUMO-1. At least 100 different proteins have been reported as targets for sumoylation [14]–[17]. Analogous to conjugation mechanisms of Ub/Ubls, SUMO is attached to target proteins following sequential activation by E1, E2 and in most cases, E3 enzymes [18]. Following activation of the SUMO precursor [4], the E1 enzyme Aos1/Uba2 and SUMO form a thioester bond. The SUMO thioester is next transferred to the active cysteine of Ubc9, the single known E2 enzyme of the sumoylation pathway [1], [4], [18]. Then SUMO is transferred from E2 to a target protein lysine residue. E3 enzymes that ensure target specificity and increase reaction efficiency usually mediate this step (Figure 1). Among the sumoylation targets, RanGAP1, p53 and IκBα are modified without an E3 ligase *in vitro*, although the reaction rates are slower compared to E3-mediated conjugation [1]. E2 ligase Ubc9 is essential [1], [19] and conserved [1]. It recognizes a consensus sumoylation motif, “Ψ-K-x-D/E”, where Ψ represents a hydrophobic residue, K is the SUMO acceptor lysine, x is any amino acid and D/E is an acidic residue [4]. The E2 ligase also interacts with E3 enzymes during the transfer of SUMO to targets [20]. In addition to the consensus sumoylation motif, sumoylation target RanGAP1 has a second contact surface with the E2 ligase Ubc9, which is thought to be responsible for the higher efficiency of modification compared to other substrates [4]. A fragment of the E3 enzyme RanBP2, consisting of the IR1-M-IR2 domains is sufficient for E3 activity *in vivo* and *in vitro* [18]. Moreover, IR1-M and M-IR2 constructs are also functional with IR1-M being the catalytic core domain [20]–[22]. The activity of the E3 fragment indicates that E3 exerts its catalytic effect by altering the structural properties of the E2-SUMO complex, increasing the affinity of the complex for specific protein targets, rather than by forming direct target interactions [21]. The crystal structure of the SUMO-RanGAP1-Ubc9-RanBP2 complex supports this idea [20]. Recent work also shows that E3 ligase RanBP2 prevents dissociation of SUMO from its target RanGAP1, leading to an increase in the sumoylated RanGAP1 levels [23]. Due to the strong interactions between RanGAP1 and E2, it has been a debated question whether RanBP2 exerts its E3 activity for RanGAP1 or whether it only maintains the complex at the nuclear pore complex (NPC) [4], [20].

**Figure 2:**
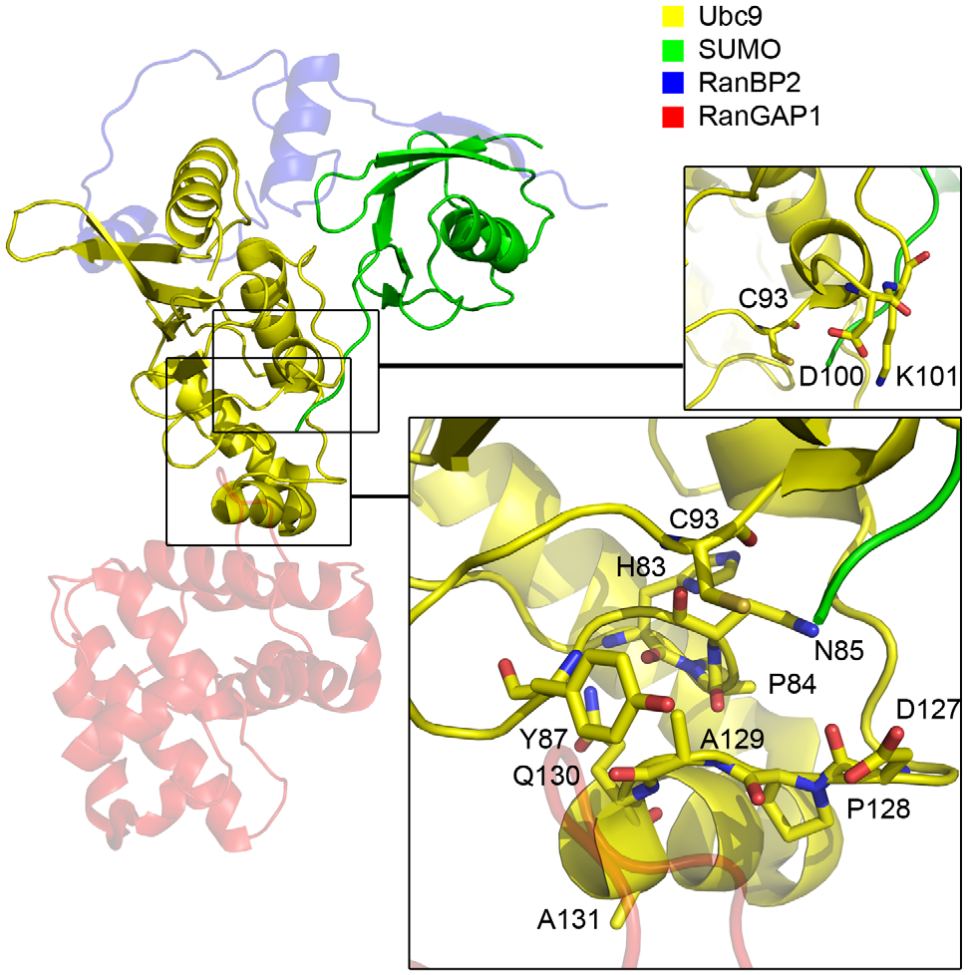
The structure of the Ubc9-SUMO-RanBP2-RanGAP1 complex [**20**]. The chains are colored as indicated in the legend. The insets highlight the residue groups that are of interest. Top: The Ubc9 loop with the Asp100 and Lys101, active Cys93 is also represented in sticks. Bottom: The residues interacting with the sumoylation motif of target proteins. Detailed version is given in Figure S5.

Our aim is to understand the mechanism through which the E3 ligase RanBP2 triggers target recognition and catalysis by E2 in sumoylation. We carried out explicit solvent molecular dynamic simulations for the E2 ligase Ubc9, SUMO, and the E2-SUMO complex with and without the E3 enzyme RanBP2. We modeled the conjugated E2-SUMO complex, in RanBP2 bound and unbound forms, based on the SUMO-RanGAP1-Ubc9-RanBP2 crystal structure (Figure 2). Our results indicate that E3 binding induces a higher population of target binding and catalysis-ready E2, restricting the conformational space of the E2-SUMO complex. We observe that RanBP2 binding enhances the correlations between the fluctuations of E2 residues involved in catalytic activity and target recognition, which implies that RanBP2 is indeed an E3 ligase for the sumoylation of the target protein RanGAP1. Our results further lead us to propose that the mechanism through which E3 ligase RanBP2 triggers E2 target recognition and catalysis in sumoylation is allostery: RanBP2 is an allosteric effector of E2 ligase Ubc9. Below, we refer to the specific proteins simulated (Ubc9, RanGAP1, RanBP2) rather than the protein functional class (E2, target protein, E3, respectively) to which they belong. These were the ones crystallized by Reverter and Lima [20].

## Results

### RanBP2 binding reduces the conformational space of the Ubc9-SUMO complex

**Figure 3:**
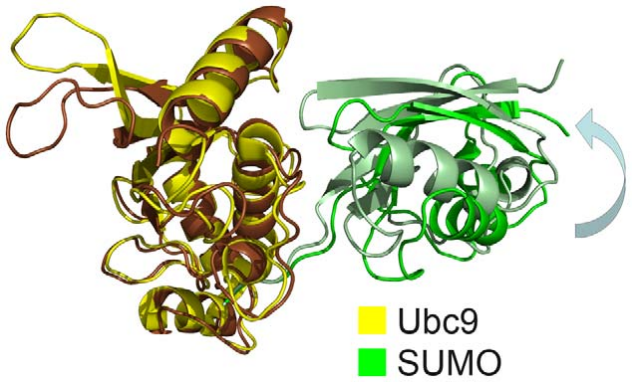
SUMO orientation change. The orientations in the crystal structure [20] (Ubc9 in yellow and SUMO in dark green) and a representative structure from the simulation trajectory (Ubc9 in brown, SUMO in light green) are aligned via the Ubc9 molecule. The orientation change in SUMO can clearly be seen. Additionally, the motion of the mobile loop of Ubc9 (Val27-Met39) can also be observed.

When simulated without RanBP2, the Ubc9-SUMO complex structure displays a significant deviation from its crystal structure. The two representative conformations from the clustering analysis of the sampled conformational space of the complexes display the change in the quaternary structure of the Ubc9-SUMO complex (Figure 3). The Ubc9-SUMO rotates and moves away from its position in the crystal structure (Figure 3). Accompanying the orientation change of SUMO, there is a minor re-organization of the hydrogen bond network in the catalytic area (Figure S1). The rmsd (root mean square deviation) of the Ubc9-SUMO complex shows that the deviation is more dramatic between 5–12 ns and stabilizes at the end of this period (Figure S2A). Yet, the monomers do not show increased deviations from their initial structures (Figure S2B, Figure S2C). This indicates that the rmsd increase of the complex structure originates from a change in the relative positions of the chains with respect to each other. On the other hand, with RanBP2, SUMO does not move or rotate but fluctuates around its original crystal conformation.

For a more quantitative measure of this orientation change, we utilize the representative structures from the clustering analysis. We carried out rmsd calculations for Ubc9 and SUMO, with alignments of Ubc9-SUMO and Ubc9-SUMO-RanBP2 complexes on the individual proteins. The results are listed in Table 1. As expected, when the proteins are aligned on the corresponding chains in the complex structure, the rmsd values are low; however, they show some increase when the complex is aligned on the other chain. The rmsd values for SUMO and Ubc9 are not significantly different for Ubc9-SUMO and Ubc9-SUMO-RanBP2 simulations when each is aligned on the corresponding chain in the complex. When the structures are aligned with Ubc9 as the pivot, the SUMO rmsd in the Ubc9-SUMO-RanBP2 complex increases up to less than 2 fold (representative structure 3), while the SUMO rmsd in the Ubc9-SUMO complex increases more than 4 fold (representative structure 4). This implies an overall quaternary structure change; that is, an orientation change of SUMO in the Ubc9-SUMO complex, which is not observed in the Ubc9-SUMO-RanBP2 complex. An additional set of simulations further validate this major orientation change (Table S1).

**Table 1:** RMSD with chain based alignments.

Ubc9-SUMO-E3 complex	Ubc9 rmsd (Å)	SUMO rmsd (Å)	
	aligned on Ubc9	aligned on SUMO	aligned on Ubc9
Representative Structure 1	1.28	0.95	1.80
Representative Structure 2	1.31	1.42	2.25
Representative Structure 3	1.19	1.30	2.47
Representative Structure 4	1.23	1.60	2.75

Ubc9-SUMO complex	Ubc9 rmsd (Å)	SUMO rmsd (Å)	
	aligned on Ubc9	aligned on SUMO	aligned on Ubc9
Representative Structure 1	1.34	1.45	1.94
Representative Structure 2	1.00	1.16	2.97
Representative Structure 3	1.23	1.46	2.63
Representative Structure 4	1.00	1.27	5.30
Representative Structure 5	2.15	1.41	5.49
Representative Structure 6	1.32	1.37	4.93

**Figure 4:**
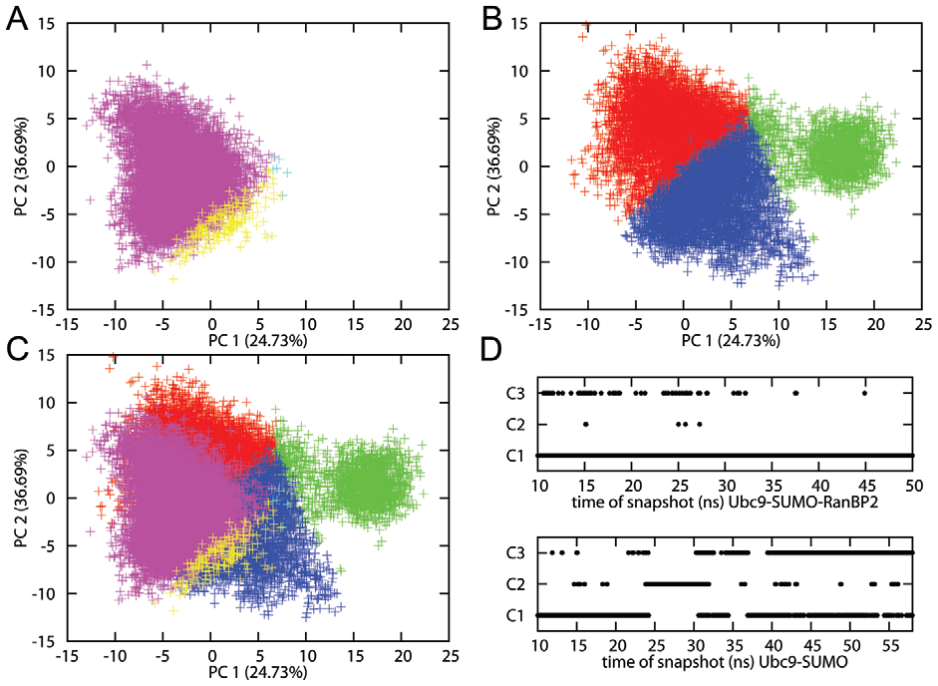
Projection of Ubc9 conformations on principal components. The PCs are given in Å. The proportion of all trajectory accounted for by the PCs up to current PC is given in parenthesis on each axis. (A–C) x-axis is PC1, y-axis PC2. (A) Ubc9 conformations from the Ubc9-SUMO-RanBP2 simulation, projected on PC1 and PC2. Members of cluster 1 in magenta, cluster 2 in cyan, cluster 3 in yellow. (B) Ubc9 conformations from Ubc9-SUMO simulation, projected on PC1 and PC2. Members of cluster 1 in red, cluster 2 in green, cluster 3 in blue. (C) Merged plot of A and B, with same color coding. (D) The distribution of cluster members in time, x-axis time, y-axis cluster number. Upper lane is Ubc9-SUMO-RanBP2, and lower lane is Ubc9-SUMO.

Along with the limitation of SUMO orientation, E3 binding restricts the conformational space of Ubc9. We combined the Ubc9 conformations from the simulations of the Ubc9-SUMO and Ubc9-SUMO-RanBP2 complexes, eliminating the initial 10 ns from each simulation, and clustering the remaining conformations. In the three resulting clusters, nearly all the Ubc9 conformations from the Ubc9-SUMO-RanBP2 complex are in one cluster, and the Ubc9 conformations from the Ubc9-SUMO complex are distributed among all three clusters. The distributions of the Ubc9 conformations are given in Table 2. The time distribution of cluster members displayed in Figure 4D shows that Ubc9 from the Ubc9-SUMO complex samples conformations from the Ubc9-SUMO-RanBP2 complex throughout the whole simulation time. The distribution of the conformations among the clusters shows that RanBP2 binding restricts the conformational space of Ubc9. To further validate this restriction, we projected the Ubc9 conformational space from the Ubc9-SUMO and Ubc9-SUMO-RanBP2 complexes on the principal components (Figure 4A–C, Figure S4). The projections and the clustering analysis demonstrate the restriction of Ubc9 conformational space upon RanBP2 binding.

**Table 2:** Clustering analysis of Ubc9 conformations from the joined ensemble.

	Percentage of ensemble belonging to cluster			
	Cluster 1	Cluster 2	Cluster 3	Total
Ubc9 from Ubc9-SUMO-E3 complex	97.97	0.06	1.97	100.00
Ubc9 from Ubc9-SUMO complex	48.19	15.07	36.74	100.0

**Figure 5:**
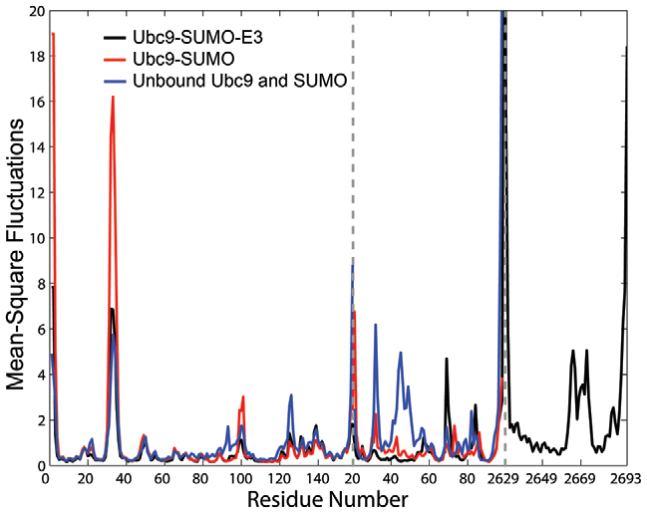
Mean square fluctuations from unbound Ubc9 (blue), unbound SUMO (blue), Ubc9-SUMO complex (red) and Ubc9-SUMO-RanBP2 complex (black) simulations. The x-axis gives the residues numbers for each chain, where the dashed lines on the figure mark the separations between chains. Although coded in the same color, the results for unbound Ubc9 and unbound SUMO are from separate simulations, details of which are given in Methods and Text S1.

The mean-square fluctuations (msf) of the proteins in their unbound states (Ubc9 and SUMO only), and in the complexes are given in Figure 5. Ubc9 Cys93 is the active cysteine, and residues from Asn124-Pro128 are part of the loop region that is in contact with the tetrapeptide motif of the sumoylation targets [4]. The fluctuations of Cys93 and Asn124-Pro128 are restricted in both Ubc9-SUMO and Ubc9-SUMO-RanBP2 complexes, as compared to the unbound state of Ubc9 (Figure 5). Thus, the catalytic residue and the residues maintaining the structural integrity around the catalytic residue of Ubc9 already display a reduced mobility in the Ubc9-SUMO complex. In terms of reduced conformational states of the catalytic region, Cys93 and Asn124-Pro128, the RanBP2 binding does not stabilize further these regions. This reduction in Ubc9 mobility is a direct result of SUMO binding. Ubc9 residues Val27-Met39 comprise the loop between β-sheets that serve as RanBP2 binding sites [20]. These residues already display high fluctuations in the isolated state, but their mobilities are allosterically further enhanced by SUMO binding. RanBP2 binding reduces the fluctuations to the values in the unbound state of Ubc9 (Figure 5). These residues also show cooperative fluctuations with RanBP2 binding sites on SUMO (discussed below).

The mobility of residues Glu98-Asp102 of Ubc9 increase upon SUMO binding, and again decrease upon RanBP2 binding (Figure 5). Residues Asp100 and Lys101 take part in target recognition, interacting with the approaching RanGAP1 [24], [25]. RanBP2 does not have direct contacts with this loop. The fluctuations of Asp100 and Lys101 in the Ubc9-SUMO-RanBP2 complex are lower than in the unbound Ubc9, whereas their fluctuations in the Ubc9-SUMO complex are higher than in the unbound Ubc9. This suggests that the restricted fluctuations of Asp100 and Lys101 are the outcome of E3 binding. On the whole, the reduced mobility of this region upon RanBP2 binding is consistent with the proposed allosteric effect of RanBP2 on the Ubc9 target recognition [20], [21]. Although the fluctuations of some SUMO residues demonstrate dramatic changes amongst all three (the two complex and one isolated) states, most of these do not coincide with functional residues. The high mobility of the unbound SUMO structure (see Text S1) necessitates further experimental evidence.

### The allosteric effect of RanBP2 on Ubc9 in sumoylation: Restriction of Asp100-Lys101 orientational motion

**Figure 6:**
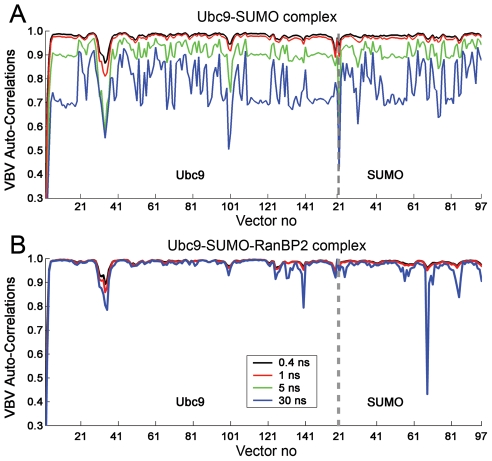
Auto-correlations of the backbone bond vectors (virtual bond vectors between successive alpha carbons). (A) The auto-correlations for Ubc9-SUMO-RanBP2 complex with time delay of 0.4 ns (black), 1 ns (red) and 30 ns (blue). (B) The auto-correlations for Ubc9-SUMO complex with time delay of 0.4 ns (black), 1ns (red), 5 ns (green) and 30 ns (blue). At time delay of 30 ns, there is overall loss of correlations in Ubc9-SUMO complex in comparison to the high correlations observed for Ubc9-SUMO-RanBP2. The correlations with 5 ns delay present first regions to loose correlations as around vector 30 and vector 101 of Ubc9.

The time delayed auto-correlations of the backbone bond vectors is a measure of their orientational freedom. They provide information on the time dependent changes in the orientations of the backbone bonds. Here, the backbone bond vector refers to the virtual bond vector between two successive Cα atoms (see Methods, Text S1). A backbone bond vector will be closely correlated with the same vector calculated after a short time interval. As the time delay increases, the correlation between the vectors will decrease. High auto-correlation of a backbone bond vector indicates a restricted orientational freedom for the backbone bond. In the Ubc9-SUMO-RanBP2 complex, the correlations of backbone bonds are not lost with time delays as high as 30 ns, with almost all virtual bond vectors having auto-correlations above 0.9, unlike the Ubc9-SUMO complex (Figure 6). This indicates that RanBP2 binding restricts the orientational freedom of Ubc9-SUMO residues. Upon RanBP2 binding, the backbone vectors between Glu99-Asp100 and Asp100-Lys101 of Ubc9 display the most significant restraint in their orientational behavior (Figure 6), particularly compared to the other loops of Ubc9. Residues Asp100-Lys101 are not in close vicinity to the RanBP2 binding regions or the catalytic region of Ubc9. The average distances of Asp100 to Cys93 throughout the Ubc9-SUMO and Ubc9-SUMO-RanBP2 simulations are 13.25 Å and 12.82 Å, respectively. Similarly, the average distances between Lys101 and Cys93 throughout the Ubc9-SUMO and Ubc9-SUMO-RanBP2 simulations are 11.54 Å and 9.83 Å, respectively. Asp100 and Lys101 of Ubc9 are known to be important for target recognition and functional defects have been observed for Ubc9 upon mutations of these residues [24], [25]. Restriction of the mobility and orientational freedom of these residues upon RanBP2 binding can hinder a pre-organization of the target binding site on Ubc9-SUMO leading to a shift of the conformational ensemble [26] of Ubc9. Since these residues are far from the RanBP2 binding site on Ubc9, the rigidification of the 99–102 loop on Ubc9 is allosterically induced by RanBP2 binding.

The Ubc9 residues Asn121-Ala131 interact with the consensus sumoylation motif and Glu132-Arg141 are important for specific RanGAP1 target binding [4], [25], [27]. The orientational freedom of the bond vectors Pro128-Ala129, Gln130-Ala131, Glu132-Ala133 and Ala133-Tyr134 are significantly reduced when bound to RanBP2. Yet, the bond vectors Asp127-Pro128 and Ala129-Gln130 already have a restricted orientational freedom in Ubc9-SUMO, together with the low msf (Figure 5). Nevertheless, RanBP2 allosterically affects the dynamics of the Ubc9 regions that interact with RanGAP1 and facilitates the RanGAP1 recognition. Other than residues around Asp100, Figure 6 points to altered orientational freedom in the Ubc9 region around Lys30. This region is part of the Val27-Met39 loop, which is highly mobile in the Ubc9-SUMO complex and displays a reduced mobility upon RanBP2 binding.

### RanBP2 binding stabilizes the correlations of Ubc9 residues functional in catalytic activity and specific target recognition

**Figure 7:**
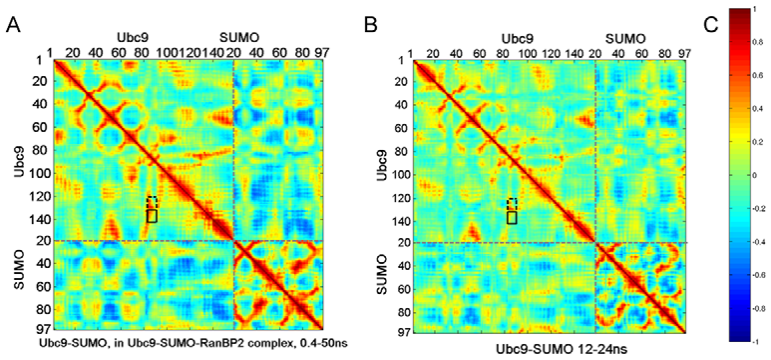
Correlations between fluctuations of residues. (A) Correlations of Ubc9-SUMO from Ubc9-SUMO-RanBP2 overall trajectory. (B) Correlations of Ubc9-SUMO from Ubc9-SUMO trajectory between 12–24 ns of simulation time. In both A and B, the dashed rectangle surrounds the correlations between His83-Ser89 Asn121-Ala131 of Ubc9, the solid rectangle surrounds the correlations between His83-Ser89 and Ala131-Arg141 of Ubc9. (C) The color bar indicating the correlations for both A and B.

The Ubc9-SUMO-RanBP2 complex has correlated fluctuations between Ubc9 regions His83-Ser89 and Asn121-Arg141. Residues His83-Ser89 include the HPN (His83-Pro84-Asn85) motif that has a structural role, maintaining the hydrogen-bonding networks around the catalytic site of Ubc9 and assisting in orienting the SUMO C-terminal Gly-Gly motif [4]. Tyr87 is in contact distance with the sumoylation motif. Ubc9 residues Asn121-Ala131 interact with the sumoylation motif of the targets, and Glu132-Arg141 play a role in the recognition of the sumoylation target RanGAP1 [4], [25], [27]. Mutations of Glu132 and Tyr134 reduce the efficiency of RanGAP1 sumoylation; whereas mutations of Asn85 and Tyr87 reduce the sumoylation efficiency for all targets [4], [25], [27]. The correlated fluctuations observed in the Ubc9-SUMO-RanBP2 complex between residues His83-Ser89 and Asn121-Ala131 are still preserved in the Ubc9-SUMO complex. However, in contrast to the stable correlations in Ubc9-SUMO-RanBP2, in the absence of RanBP2, the correlations between residues His83-Ser89 and the additional Glu132-Arg141 binding surface, fluctuate (Figure 7, Figure S3). Analysis of the Ubc9-SUMO trajectory over the time windows suggested by the clustering (see Methods, Text S1, Figure S2A and Table S2), shows that the correlation between His83-Ser89 and the Glu132-Arg141 is lost between 12–24 ns (20.9 percent of simulation time), and still weak between 24–31 ns (12.2 percent of simulation time) with respect to the Ubc9-SUMO-RanBP2 (Figure S3).

Coinciding with the orientation change of SUMO, the correlations between the fluctuations of the Ubc9 region including the mobile loop, Val27-Glu42, and the rest of the Ubc9 residues, become more negative. Furthermore, the same region, Val27-Glu42, displays cooperative fluctuations with SUMO residues Phe36-Leu47 and Asp73-Ile88. The two regions of SUMO are either on the β-sheets packed against RanBP2 or in the vicinity of these β-sheets in RanBP2 binding site in SUMO. Upon RanBP2 binding these correlations become more prevalent. The loop in Ubc9 and those SUMO regions are spatially far away. When using the centers of mass, the average distance between Val27-Glu42 loop of Ubc9 and Phe36-Leu47 of SUMO is ∼45.0 Å and 47.6 Å for Ubc9-SUMO and Ubc9-SUMO-RanBP2 simulations. Similarly, the average distance between the Val27-Glu42 loop and the Asp73-Ile88 of SUMO is 44.8 Å for Ubc9-SUMO and 45.6 Å for the Ubc9-SUMO-RanBP2 complex simulations.

Alterations in the correlated fluctuations of regions that are not listed above are observed from the correlation maps. In many of these cases, although an overall change is observed in a region in the vicinity of a key residue, the correlation of the residue itself is conserved. One such example is the reduction in the correlated fluctuations of Ubc9 residues around position 70, with both residues around 100 and the first 10 residues of Ubc9. Lys74, the key residue in this region which contacts the consensus sumoylation motif in targets [4], conserves its correlations.

To summarize, the correlated fluctuations among the residues responsible for the structural integrity of the complex catalytic region (His83-Ser89) and the residues that interact with the sumoylation motif of the targets (Asn121-Ala131) are stable for both Ubc9-SUMO and Ubc9-SUMO-RanBP2 complexes. On the other hand, the correlated fluctuations between His83-Ser89 and residues playing a role in selective target recognition (Glu132-Arg141) are less stable in the Ubc9-SUMO complex. These correlations are allosterically stabilized upon RanBP2 binding. Further, the correlations between RanBP2 binding sites on Ubc9 and on SUMO pre-exist RanBP2 binding; yet, as expected, they are enhanced upon binding. Our results are summarized in Table 3.

**Table 3:** Summary of key residues and related observations.

Ubc9 Residue(s)	Function	Observations from Dynamics
Cys93	Catalytic cysteine [1], [4], [18].	Restricted in both Ubc9-SUMO and Ubc9-SUMO-RanBP2 complexes relative to unbound Ubc9 structure.
Val27 – Glu42	Includes loop between β-sheets serving as RanBP2 binding sites [20].	The loop has high mobility in unbound state. The mobility is further increased in Ubc9-SUMO complex. RanBP2 binding reduces mobility to unbound Ubc9 level.
		In Ubc9-SUMO complex, displays correlated fluctuations with RanBP2 binding regions of SUMO, which are spatially distant.
His83 – Ser89	Maintains hydrogen bonding networks around catalytic site, assists orientation of SUMO C-terminal Gly-Gly motif [4], [25], [27].	Correlated fluctuation with Asn121-Ala131 protected in Ubc9-SUMO and Ubc9-SUMO-RanBP2 complexes.
		Correlated fluctuation with Glu132-Arg141 are mostly lost in Ubc9-SUMO complex, but enhanced by RanBP2 binding.
Asp100 – Lys101	Target recognition [24], [25].	Increased msf in Ubc9-SUMO complex relative to unbound state. Fluctuations reduced below unbound state in Ubc9-SUMO-RanBP2 complex.
		Increased orientational freedom in Ubc9-SUMO complex, rigidification in Ubc9-SUMO-RanBP2 complex.
Asn124 – Pro128	Loop in contact with consensus sumoylation motif in targets [4].	Restricted in both Ubc9-SUMO and Ubc9-SUMO-RanBP2 complexes relative to unbound Ubc9 structure.
Glu132 – Arg 141	RanGAP1 specific target binding [4], [25], [27].	Orientational freedom of region reduced in Ubc9-SUMO-RanBP2 complex.
		Correlations with His83 – Ser89 are mostly lost in Ubc9-SUMO complex, but enhanced by RanBP2 binding.

## Discussion

Two mechanisms have been proposed [1], [4] for RanGAP1 target sumoylation (Figure 1). In both, the first step involves Ubc9 conjugation to SUMO. In the first mechanism Ubc9-SUMO binds to the target and an E3 ligase, whereas in the second Ubc9-SUMO can bind and sumoylate the target without an E3 ligase. In order to understand the role of E3 enzymes in the pre-organization of the Ubc9-SUMO complex in the mechanism of sumoylation, we simulated the Ubc9-SUMO complex with and without the E3 ligase RanBP2. Based on the two conformational ensembles with and without E3, Ubc9 is already largely pre-organized for target binding and catalysis [28], [29], yet the orientation of SUMO differs in Ubc9-SUMO and Ubc9-SUMO-RanBP2 complexes. Analysis of the conformational ensembles of Ubc9, SUMO, Ubc9-SUMO, and Ubc9-SUMO-RanBP2 revealed that RanBP2 binding allosterically shifts the equilibrium of Ubc9 conformations, restricts SUMO orientation, and enhances the populations of the pre-organized conformational states [26], [28], [29], [30]. RanBP2 binding reduces the conformational space sampled by Ubc9 (Figure 4, Figure S4). At the same time, the RanBP2 binding allosterically reduces the mobility and the orientational freedom of the Ubc9 residues, with the effect being particularly dramatic around Asp100 and Lys101 target recognition residues [24], [25]. RanBP2 binding allosterically enhances the correlated fluctuations of Ubc9, mainly for residues around the catalytic and specific target recognition sites. The RanBP2 binding sites on Ubc9 and on SUMO which are spatially far apart display correlated fluctuations even in the absence of E3; however upon E3 binding these correlations get stronger.

RanBP2 was proposed to limit the available conformations of the Ubc9-SUMO complex and prevent non-productive conformations [20], [21]. Our simulations demonstrate that upon RanBP2 removal there is a change in the relative position of SUMO with respect to Ubc9, yet the removal of RanGAP1 does not affect SUMO's position. Furthermore, in the absence of RanBP2, RanGAP1 is not sufficient to prevent SUMO's position change (unpublished data). This leads us to propose a mechanism where RanBP2 binding to the Ubc9-SUMO complex triggers SUMO's stabilization in a catalytically efficient orientation, with a subsequent target binding. This is consistent with RanBP2 enhanced allosteric effects on Ubc9's Asp100-Lys101, the specific target recognition regions, and the correlated fluctuations of RanBP2 binding sites in Ubc9 and SUMO in the absence of RanBP2.

A correlation between the fluctuations implicates a network of interacting residues. It is highly plausible to expect an overlap of such a network with functional residues. We observe coupled fluctuations displaying changes between RanBP2 bound and unbound states. Ubc9 residues Lys74, Tyr87, Ser89, Thr91, Cys93, Asp127, Pro128, Ala129, Gln130 and Ala131, interact with the consensus sumoylation tetrapeptide motif in most sumoylation targets [4]. The correlations between Ubc9 residues responsible for the structural integrity of the complex catalytic region (His83-Ser89) and residues Asn121-Ala131 are conserved in both Ubc9-SUMO and Ubc9-SUMO-RanBP2. Additionally, there is a second contact surface on Ubc9 (Glu132-Asn140), specific for the sumoylation target RanGAP1. Because of the strong interactions between Ubc9 and RanGAP1 [4] through this additional binding region, the need for E3 activity for sumoylation of this target has not been apparent [4], [20]. The enhanced correlations between Ubc9 residues His83-Ser89 and Glu132-Arg141 upon RanPB2 binding indicate that this additional binding surface is linked to the catalytic activity. The stronger correlations (Figure 7) suggest that RanBP2 increases the efficiency of this additional target binding surface on Ubc9.

The mobilities of the catalytic Cys93, Asp127 and Pro128 of Ubc9, which contact the consensus sumoylation tetrapeptide motif in sumoylation targets [4], are reduced with the SUMO binding, and RanBP2 binding does not lead to a further stability of these residues. Additionally, we observe the conservation of the correlations between His83-Ser89 and Asn121-Ala131 of Ubc9, and the restrictions of the orientational freedom for Asp127-Pro128 and Ala129-Gln130 in the Ubc9-SUMO complex without RanBP2. The pre-existing tendencies of these residues which have roles in catalysis and target recognition in the absence RanBP2 may indicate why Ubc9 can function without the aid of an E3 enzyme. Nevertheless, the correlations between other functional regions, such as between His83-Ser89 and Glu132-Arg141, are enhanced with a restriction in the conformational freedom of Ubc9-SUMO with RanBP2. Indeed, the restriction in the conformational space of Ubc9-SUMO with RanBP2 was suggested as a means of increasing sumoylation efficiency [20], [21].

The mobility and orientational freedom of the Ubc9 Val27-Met39 loop is affected by RanBP2 binding. This loop displays correlated fluctuations with SUMO residues Phe36-Leu47 and Asp73-Ile88, which are in close vicinity to the RanBP2 binding sites. Together, these point to a sequence of events in the formation of the complex which translate to SUMO binding to Ubc9, followed by RanBP2 binding. From a mechanistic point of view, SUMO binds to Ubc9, allosterically enhancing the mobility of the Val27-Met39 loop and residues Asp100-Lys101, with the Val27-Met39 loop coordinated with the RanBP2 binding sites on SUMO. Next RanBP2 binds to the Ubc9-SUMO complex, moving SUMO closer to the target binding orientation, restricting the Ubc9 conformations, and at the same time RanBP2 induces allosteric changes in Ubc9 target recognition and catalytic sites. The changes in the network of hydrogen-bonds between Ubc9 and SUMO between the RanBP2 bound and unbound states appears to be the driving force behind the orientation limitation of SUMO induced by RanBP2 (Figure S1). The limitations imposed on the orientational freedom of Asp100-Lys101 of Ubc9, and increased correlations between Ubc9 catalytic site residues (His83-Ser89) and specific target recognition residues (Glu132-Asn140) [4], [20] induced by RanBP2 binding, support the proposed mechanism.

Here we propose that the role of E3 ligase RanBP2 in sumoylation is to restrict the conformational freedom of the E2-SUMO complex and to increase the reaction efficiency via allosteric effects [31] on the E2 Ubc9. RanBP2 binding to the E2-SUMO complex limits the accessible conformations of Ubc9 and the orientational space of the Ubc9 and SUMO monomers. In particular, the positional and orientational freedom of Ubc9 residues Asp100-Lys101, important for target recognition [24], [25] is restricted upon RanBP2 binding. RanBP2 binding stabilizes the correlations among Ubc9 residues that are functional in specific target recognition (Glu132-Asn140) and catalytic activity (His83-Ser89) [4], [20]. Mechanistically, the correlations we observe in the dynamics of the E2-SUMO complex argue for such sequence of events in sumoylation and provide an explanation to the question of why sumoylation can also take place in the absence of an E3, although with lower efficiency.

## Methods

### Molecular dynamics protocol

Molecular dynamics (MD) simulations are run on Ubc9, SUMO, and the Ubc9-SUMO and Ubc9-SUMO-RanBP2 complexes, using *Amber 8* [32], [33]. Software and simulation parameter details are provided in the Text S1.

The structures of human Ubc9 (PDB code: 1A3S) and human SUMO-1 (PDB code: 1A5R) provide a base to analyze the effects of RanBP2 binding to the Ubc9-SUMO complex. The modeled structures for the intermediate complexes Ubc9-SUMO and Ubc9-SUMO-RanBP2 are extracted from the crystal structure of Ubc9-SUMO-RanGAP1-RanBP2 complex (PDB code: 1Z5S). In the latter complex, as SUMO is bound to the acceptor lysine of target protein RanGAP1, the thioester bond between Ubc9 active cysteine and SUMO C-terminal glycine is modeled in the co-crystal complexes of Ubc9-SUMO and Ubc9-SUMO-RanBP2. The modeling of the thioester bond is detailed in Text S1. Two sets of simulations are carried out. In the set where detailed time window based analysis is carried out, the simulation lengths for the structures are: unbound Ubc9, 32.5 ns; unbound SUMO, 35 ns; Ubc9-SUMO complex, 58 ns; Ubc9-SUMO-RanBP2 complex, 50 ns. The second set is carried out to validate the major orientation change of SUMO observed in the Ubc9-SUMO structure in the first set of simulations. The simulation lengths for each complex or protein consider the specific characteristics of the structure, details of which are given in the Text S1.

### Clustering analysis and principal component analysis

The simulations generate a large number of different conformations of the structures, many of which may be similar. To obtain distinct representative conformations, we clustered the conformations with k-means clustering (MMTSB Toolset's *kclust* utility [34]), taking the rmsd of residue positions from the cluster centroid as the similarity measure. Rmsd values of 2 Å, 1.7 Å and 1.5 Å are tested; a smaller number of clusters appear as the rmsd increases. The rmsd is set to 1.7 Å for Ubc9-SUMO complex and 2 Å for Ubc9-SUMORanBP2 complex. For the joined conformational space analysis on Ubc9, the alignment is made on the Ubc9 conformation from the Ubc9-SUMO complex at 10 ns, and 1.7 Å cut-off is used.

The principal component analysis is carried out using the *ptraj* module of *AMBER 8.0*. The alignment of the joined ensemble, Ubc9 conformations from the Ubc9-SUMO and the Ubc9-SUMO-RanBP2 complexes, followed the same procedure as in the clustering analysis. The results are generated by projecting the Ubc9 conformations on the PC of the joined ensemble. The proportion of the eigenvalue of each PC to the sum of all eigenvalues represents the contribution of the PC to the all conformations in the trajectory. The cumulative contribution of all PCs up the PC of interest is given in the axes of Figure 4 and Figure S4.

### Residues' mean-square fluctuations and cross-correlations

The equilibration periods of the simulation (2.5 ns for Ubc9, 5 ns for SUMO, 0.5 ns for Ubc9-SUMO and 0.4 ns for Ubc9-SUMO-RanBP2) are excluded in the calculations. For the msf calculations of each chain in the complexes, the alignment is carried out on the corresponding chain only, to eliminate the poor alignment that may possibly result from the structural changes in the quaternary structure. In the case of SUMO, the flexible N-terminal tail (residues −1 to 19 in PDB 1A5R) of SUMO is eliminated for the same reason.

The normalized correlations between the fluctuations of residues, the cross-correlations, are defined by Eq. 1 as:
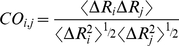
(1)Where Δ**R**_i_ and Δ**R**_j_ are the fluctuation in the position vectors, **R**_i_ and **R**_j_ of residues *i* and *j*, respectively. The cross-correlations vary in the range [−1, 1] with the lower and upper limit indicating fully anti-correlated and correlated fluctuations, respectively. The correlations are calculated for the total length of the trajectory and for the time windows defined by the clustering analysis.

### The time delayed auto-correlations of the backbone bond vectors

The backbone bond vector is defined as the normalized vector **M**_i_ from the α carbon C^α^_i−1_ of residue *i−1* to the α carbon C^α^_i_ of residue *i*. The normalized time-delayed auto-correlations of these virtual bond vectors are defined as in Eq. 2:
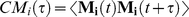
(2)Where **M**_i_ (t) and **M**_i_ (t+τ) are the virtual bond vectors of *i* at time *t* and *t+τ*, respectively. The brackets represent averages over recorded snapshots. The auto-correlations are in the range [−1, 1] with the lower and upper limit indicating fully anti-correlated and correlated virtual bonds, respectively. *τ* = 0 gives the equal-time auto-correlations, which is 1 for all virtual bond vectors. The correlations are calculated for several time delays τ, from 0 to 30 ns. The highest value of the time delay (30 ns) is selected to be slightly longer than half simulation times, for both Ubc9-SUMO-RanBP2 and Ubc9-SUMO complexes.

## Supporting Information

Text S1 Detailed methodology of the study.
(0.05 MB DOC)

Figure S1 Distances between potential hydrogen bonds. (A) Distance between alpha carbon atoms of residues Arg63 of SUMO and Glu122 of Ubc9 throughout the trajectories. Upper lane is the distance for Ubc9-SUMO-RanBP2 complex, and lower lane is the distance for Ubc9-SUMO complex. (B) Distance between α carbon atoms of residues Gln29 of SUMO and Gln111 of Ubc9 throughout the trajectories. Upper lane is the distance for Ubc9-SUMO-RanBP2 complex, and lower lane is the distance for Ubc9-SUMO complex.
(4.23 MB TIF)

Figure S2 Rmsd values for both complexes throughout the simulation. (A) The rmsd values with the alignment of the whole complex structure, throughout the simulation for Ubc9-SUMO complex. The jump in rmsd can be observed around 10 ns. The rmsd values for the same simulation, calculated by alignment of individual chains are illustrated for (B) Ubc9 and for (C) SUMO. The rmsd jump is not observed in B and C. (D) The rmsd values with the alignment of the whole complex structure, throughout the simulation for Ubc9-SUMO-RanBP2 complex.
(4.49 MB TIF)

Figure S3 Correlations of mean-square fluctuations. (A) Correlations of Ubc9-SUMO overall trajectory. (B) Correlations of Ubc9-SUMO from Ubc9-SUMO trajectory between 24–31 ns of simulation time. In both A and B, the rectangles surround the correlations between His83-Ser89 and Asn121-Ala131 of Ubc9, and correlations between His83-Ser89 and Ala131-Arg141 of Ubc9. (C) The color bar indicating the correlations for both A and B.
(2.14 MB TIF)

Figure S4 The projections of Ubc9 conformations on principal components. The projections of Ubc9 conformations from Ubc9-SUMO and Ubc9-SUMO-RanBP3 simulations are given in blue and red, respectively. The principal components are given in Å. All plots are in the range [−10∶10] in x- and y-axes. The proportion of all trajectory accounted for accounted for by the PCs up to current PC is given in parenthesis on each axis.
(1.80 MB TIF)

Figure S5 The structure of the Ubc9-SUMO-RanBP2-RanGAP1 complex [20]. This figure is a detailed version of Figure 2 of manuscript. The chains are colored as indicated in the legend. The insets highlight the residue groups that are of interest. Top left: Ubc9 mobile loop Val27 to Glu42. Bottom left: Ubc9 residues Glu132-Arg141, responsible for specific target recognition. Top right: SUMO residues Phe36 to Leu47 and Asp73 to Ile88. These regions mark the proximity of SUMO residues that pack with E3 and the also show correlated fluctuations with Ubc9 residues Val27 to Glu42. Middle right: Ubc9 catalytic Cys93, residues functional in target recognition Asp100, Lys101. Bottom right: Ubc9 HPN (His83-Pro84-Asn85) motif, has a structural role, maintains the hydrogen-bonding networks around the catalytic site of Ubc9. Ubc9 residues which interact with the consensus sumoylation motif, see text for functional details of individual residues.
(1.61 MB TIF)

Figure S6 Rmsd values for unbound Ubc9 and SUMO throughout the simulation. (A) The rmsd values of Ubc9. (B) The rmsd values for SUMO. Values for full length protein are displayed in red, values for N-terminal truncated protein are in blue. The effect of the first 21 residues of protein on calculations can be seen from the difference between two plots. The truncated values are used for comparison through text.
(0.31 MB TIF)

Table S1 RMSD with chain based alignments, second set of simulations.
(0.04 MB DOC)

Table S2 Time windows defined by the clustering analysis.
(0.03 MB DOC)

## References

[1] Melchior F (2000). SUMO-Nonclassical Ubiquitin.. Annu Rev Cell Dev Biol.

[2] Capili AD, Lima CD (2007). Taking it Step by Step: Mechanistic insights from structural studies of Ubiquitin/Ubiquitin-like protein modification pathways.. Curr Op Struct Bio.

[3] Müller S, Cartsen H, Pyrowolakis G, Jentsch S (2001). SUMO, Ubiquitin's Mysterious Cousin.. Nat Rev Mol Cell Biol.

[4] Bernier-Villamor V, Sampson DA, Matunis MJ, Lima CD (2001). Structural basis for E2 mediated SUMO conjugation revealed by a complex between ubiquitin-conjugating enzyme Ubc9 and RanGAP1.. Cell.

[5] Boddy MN, Howe K, Etkin LD, Solomon E, Freemont PS (1996). PIC, novel ubiquitin-like protein which interacts with the PML component of a multiprotein complex that is disrupted in acute promyelocytic leukaemia.. Oncogene.

[6] Mahajan R, Delphin C, Guan T, Gerace L, Melchinor F (1997). A small ubiquitin-related polypeptide involved in targeting RanGAP1 to nuclear pore complex protein RanBP2.. Cell.

[7] Matunis MJ, Coutavas E, Blobel G (1996). A novel ubiquitin-like modification modulates the partitioning of the Ran GTPase-activating protein RanGAP1 between the cytosol and the nuclear pore complex.. J Cell Biol.

[8] Okura T (1996). Protection against Fas/APO-1- and tumor necrosis factor-mediated cell death by a novel protein, sentrin.. J Immunol.

[9] Shen Z, Pardington-Purtymun PE, Comeaux JC, Moyzis RK, Chen DJ (1996). UBL1, a human ubiquitin-like protein associating with human RAD51/RAD52 proteins.. Genomics.

[10] Capili AD, Lima CD (2007). Structure and Analysis of a Complex between SUMO and Ubc9 Illustrates Features of a Conserved E2-Ubl Interaction.. J Mol Biol.

[11] Ulrich HD (2009). Ubiquitin, SUMO and the maintenance of genome stability.. DNA Repair (Amst).

[12] Mo Y, Yu Y, Theodosiou E, Ee PLR, Beck WT (2005). A Role for Ubc9 in Tumorigenesis.. Oncogene.

[13] Vigodner M, Weisburg JH, Shrivastava V, Marmor RA, Fathy J (2009). Differential expression patterns of SUMO proteins in HL-60 cancer cell lines support a role for sumoylation in the development of drug resistance.. Cell Tissue Res.

[14] Panse V-G, Hardeland U, Werner T, Kuster B, Hurt E (2004). A proteome wide approach identifies sumoylated substrate proteins in yeast.. J Biol Chem.

[15] Wohlschlegel JA, Johnson ES, Reed SI, Yates JR (2004). Global analysis of protein sumoylation in *Saccharomyces cerevisiae*.. J Biol Chem.

[16] Zhao Y, Kwon SW, Anselmo A, Kaur K, White MA (2004). Broad spectrum identification of cellular small ubiquitin-related modifier (SUMO) substrate proteins.. J Biol Chem.

[17] Zhou W, Ryan JJ, Zhou H (2004). Global analyses of sumoylated proteins in *Saccharomyces cerevisiae* induction of protein sumoylation by cellular stresses.. J Biol Chem.

[18] Tang Z, Hecker CM, Scheschonka A, Betz H (2009). Protein interactions in the sumoylation cascade–lessons from X – ray structures.. FEBS Journal.

[19] van Waardenburg RCAM, Duda DM, Lancaster CS, Schulman B-A, Bjornsti M-A (2006). Distinct Functional Domains of Ubc9 Dictate Cell Survival and Resistance to Genotoxic Stress.. Mol Cell Biol.

[20] Reverter D, Lima CD (2005). Insights into E3 ligase activity revealed by a SUMO-RanGAP1-Ubc9-Nup358 complex.. Nature.

[21] Pichler A, Gast A, Seeler JS, Dejean A, Melchior F (2002). The Nucleoprotein RanBP2 Has SUMO1 E3 Ligase Activity.. Cell.

[22] Pichler A, Knipscheer P, Saitoh H, Sixma TK, Melchior F (2004). The RanBP2 SUMO E3 ligase is neither HECT- nor RING-type.. Nat Struct Mol Biol.

[23] Zhu S, Goeres J, Sixt KM, Békés M, Zhang X-D (2009). Protection from isopeptidase-mediated deconjugation regulates paralog-selective sumoylation of RanGAP1.. Mol Cell.

[24] Tatham MH, Chen Y, Hay RT (2003). Role of two residues proximal to the active site of Ubc9 in substrate recognition by the Ubc9.SUMO-1 thiolester complex.. Biochemistry.

[25] Yunus AA, Lima CD (2006). Lysine activation and functional analysis of E2-mediated conjugation in the SUMO pathway.. Nat Struct Mol Biol.

[26] Gunasekaran K, Ma B, Nussinov R (2004). Is allostery an intrinsic property of all dynamic proteins?. Proteins.

[27] Wu PY, Hanlon M, Eddins M, Tsui C, Rogers RS (2003). A conserved catalytic residue in the ubiquitin-conjugating enzyme family.. EMBO J.

[28] Ma B, Kumar S, Tsai CJ, Nussinov R (1999). Folding funnels and binding mechanisms.. Protein Eng.

[29] Kumar S, Ma B, Tsai CJ, Sinha N, Nussinov R (2000). Folding and binding cascades: dynamic landscapes and population shifts.. Protein Sci.

[30] Tsai CJ, del Sol A, Nussinov R (2009). Protein allostery, signal transmission and dynamics: a classification scheme.. Mol BioSyst.

[31] Tsai CJ, del Sol A, Nussinov R (2008). Absence of a change in shape does not imply that allostery is not at play.. J Mol Bio.

[32] Case DA, Darden TA, Cheatham TE, III Simmerling CL, Wang J (2004). AMBER 8, University of California, San Francisco..

[33] Case DA, Cheatham TE, Darden T, Gohlke H, Luo R (2005). The Amber biomolecular simulation programs.. J Computat Chem.

[34] Feig MJ, Karanicolas J, Brooks CL (2004). MMTSB Tool Set: enhanced sampling and multiscale modeling methods for applications in structural biology.. J Mol Graph Model.

