# Supplementary material for: A Mechanistic View of the Role of E3 in Sumoylation
Source: PLoS Comput Biol. 2010 Aug 26;6(8):e1000913. doi: 10.1371/journal.pcbi.1000913 (PMC2928739; doi:10.1371/journal.pcbi.1000913)
Supplement: Text S1 — Detailed methodology of the study. (0.05 MB DOC) [file pcbi.1000913.s001.doc]

**Supporting Information**

**The Molecular Dynamics Software and Simulation Parameters**

Molecular dynamics (MD) simulations are run on all structures using *MD package Amber 8* (1, 2). The forcefield ff03 was used, with explicit solvent, using truncated octahedron box and TIP3P water model (3). Periodic boundary conditions at constant pressure are utilised, with isotropic position scaling. Electrostatic energy of a periodic box is calculated using The Particle Mesh Ewald (PME) method (4). The nonbonded cutoff is selected as 9 Å. The SHAKE algorithm is used and bond interactions involving H-atoms are omitted (5). After the minimization, the velocities are generated first at 10 K from a Maxwellian distribution, the temperature was gradually raised to, and maintained at 300 K by the Berendsen weak-coupling algorithm (6). The coordinates of the simulation are recorded at every 0.1 picosecond.

The simulation lengths are selected for each simulated structure individually. Initially, we started with the aim of simulating complex structures for 50 ns and unbound structures for 35 ns. The unbound Ubc9 structure is a stable protein, with low rmsd values (Figure S6A), therefore, a relatively shorter simulation time (32.5 ns) is considered sufficient for this protein. The unbound SUMO is a protein with high fluctuations in its N-terminal loop which includes first 21 residues (from -1 to 19) in the crystal structure (PDB: 1A5R). The rmsd is around 3 A when this loop is not considered, whereas it increases up to 10 A, due to poor alignment of the protein caused by high mobility of the N-terminal loop, when the whole structure is considered (Figure S6B). Because of this region, we decided to keep the simulation time of this structure at the length of 35 ns, to have a larger set of conformation samples. The Ubc9-SUMO complex is simulated longer than the remaining structures, due to the discussed orientation change (58ns). We increased the simulation time to see if the structure will go back to it original orientation. The Ubc9-SUMO-RanBP2 complex is simulated for the initially determined length of 50 ns. A second set of simulations were run to validate the major orientation change of SUMO. This set comprised of simulations of Ubc9-SUMO and Ubc9-SUMO-RanBP2 for 20 ns each, with the above parameters.

**Modeling of the Thioester Bond**

A thioester bond between Cys93 of Ubc9 and C-terminal Gly97 of SUMO is modelled. For defining the bond between these residues, first residue types with desired atoms are defined for both residues. The parameters are either taken from the classical residue types in Amber, or generated using *General Amber Force Field* (gaff) in conjunction with antechamber program (1, 2, 7). Along the way, the program *Gaussian* is used for geometry minimization. For the Cys residue in thioester bond, the atom types and parameters, the point charges, van der Waals parameters, are taken from the residue type “Cyx”. The residue Gly97 is an C-terminal residue and Amber package automatically includes the -COOH group to this residue. A new residue type “Glb”, standing for Glycine-bonded, is defined, which does not include the -COOH group. The atom types and parameters, the point charges, van der Waals parameters, are taken from the residue type “Gly” for this new residue type. For gaff and antechamber to be able to recognize the bond between these defined residues, it is necessary to reduce their distance in the crystal structure. Using the *Gaussian* method (8-11) B3LYP/6-31+G**, on the di-peptide constructed from the generated residues, the residues are brought into close distance. The necessary parameters are generated by gaff and antechamber using the minimized peptide. The residues Cyx93 and Glb97 are bonded by tleap in Amber. Stretch, bend and torsion parameters generated by the method described above are used for the defined bond.

**References**

1. Case DA, Darden TA, Cheatham TE, III Simmerling CL, Wang J, Duke RE, Luo R, Merz KM, Wang B, Pearlman DA, Crowley M, Brozell S, Tsui V, Gohlke H, Mongan J, Hornak V, Cui G, Beroza P, Schafmeister C, Caldwell JW, Ross WS, Kollman PA (2004) AMBER 8. University of California, San Francisco.
2. Case DA, Cheatham 3rd TE, Darden T, Gohlke H, Luo R, et al. (2005) The Amber biomolecular simulation programs. J Computat Chem 26: 1668-1688.
3. Jorgensen WL, Chandrasekhar J, Madura JD, Impey RW, Klein ML (1983) Comparison of simple potential functions for simulating liquid water. J Chem Phys 79: 926-935.
4. Essman U *et al* (1995) A smooth Particle Mesh Ewald method. J Chem Phys 103: 8577-8593.
5. Ryckaert JP, Ciccotti G, Berendsen HJC (1977) Numerical integration of the Cartesian equations of motion of a system with constraints: Molecular dynamics of n-alkanes. *J Comput Phys* 23: 327-341.
6. Berendsen HJC, Postma JPM, Van Gunsteren WF, DiNola A, Haak JR (1984) Molecular dynamics with coupling to an external bath. J Chem Phys 81: 3684-3690.
7. Wang J, Wolf RM, Caldwell JW, Kollman PA, Case DA (2004) Development and testing of a general amber force field. J Computat Chem 25: 1157-1174.
8. Becke AD (1988) Density-functional exchange-energy approximation with correct asymptotic behavior. Phys Rev 38: 3098–3103.
9. Becke AD (1993) A new mixing of Hartree–Fock and local density-functional theories. J Chem Phys 98: 1372–1377.
10. Becke AD (1993) Density-functional thermochemistry. III. The role of exact exchange. J Chem Phys 98: 5648–5652.
11. Lee C, Yang W, Parr RG (1988) Development of the Colle-Salvetti correlation-energy formula into a functional of the electron density Phys Rev 37: 785–789.

**Supporting Figure Legends**

Supporting Figure 1. Distances between potential hydrogen bonds. (A) Distance between alpha carbon atoms of residues Arg63 of SUMO and Glu122 of Ubc9 throughout the trajectories. Upper lane is the distance for Ubc9-SUMO-RanBP2 complex, and lower lane is the distance for Ubc9-SUMO complex. (B) Distance between α carbon atoms of residues Gln29 of SUMO and Gln111 of Ubc9 throughout the trajectories. Upper lane is the distance for Ubc9-SUMO-RanBP2 complex, and lower lane is the distance for Ubc9-SUMO complex.

Supporting Figure 2. Rmsd values for both complexes throughout the simulation. (A) The rmsd values with the alignment of the whole complex structure, throughout the simulation for Ubc9-SUMO complex. The jump in rmsd can be observed around 10 ns. The rmsd values for the same simulation, calculated by alignment of individual chains are illustrated for (B) Ubc9 and for (C) SUMO. The rmsd jump is not observed in B and C. (D) The rmsd values with the alignment of the whole complex structure, throughout the simulation for Ubc9-SUMO-RanBP2 complex.

Supporting Figure 3. Correlations of mean-square fluctuations. (A) Correlations of Ubc9-SUMO overall trajectory. (B) Correlations of Ubc9-SUMO from Ubc9-SUMO trajectory between 24-31 ns of simulation time. In both A and B, the rectangles surround the correlations between His83-Ser89 and Asn121-Ala131 of Ubc9, and correlations between His83-Ser89 and Ala131-Arg141 of Ubc9. (C) The color bar indicating the correlations for both A and B.

Supporting Figure 4. The projections of Ubc9 conformations on principal components.

The projections of Ubc9 conformations from Ubc9-SUMO and Ubc9-SUMO-RanBP3 simulations are given in blue and red, respectively. The principal components are given in Ǻ. All plots are in the range [-10:10] in x- and y-axes. The proportion of all trajectory accounted for accounted for by the PCs up to current PC is given in parenthesis on each axis.

Supporting Figure 5. The structure of the Ubc9-SUMO-RanBP2-RanGAP1 complex [21]. This figure is a detailed version of Figure 2 of manuscript. The chains are colored as indicated in the legend. The insets highlight the residue groups that are of interest. Top left: Ubc9 mobile loop Val27 to Glu42. Bottom left: Ubc9 residues Glu132-Arg141, responsible for specific target recognition. Top right: SUMO residues Phe36 to Leu47 and Asp73 to Ile88. These regions mark the proximity of SUMO residues that pack with E3 and the also show correlated fluctuations with Ubc9 residues Val27 to Glu42. Middle right: Ubc9 catalytic Cys93, residues functional in target recognition Asp100, Lys101. Bottom right: Ubc9 HPN (His83-Pro84-Asn85) motif, has a structural role, maintains the hydrogen-bonding networks around the catalytic site of Ubc9. Ubc9 residues which interact with the consensus sumoylation motif, see text for functional details of individual residues.

Supporting Figure 6. Rmsd values for unbound Ubc9 and SUMO throughout the simulation. (A) The rmsd values of Ubc9. (B) The rmsd values for SUMO. Values for full length protein are displayed in red, values for N-terminal truncated protein are in blue. The effect of the first 21 residues of protein on calculations can be seen from the difference between two plots. The truncated values are used for comparison through text.
