# Supplementary figures and images for: A Mechanistic View of the Role of E3 in Sumoylation
Source: PLoS Comput Biol. 2010 Aug 26;6(8):e1000913. doi: 10.1371/journal.pcbi.1000913 (PMC2928739; doi:10.1371/journal.pcbi.1000913)

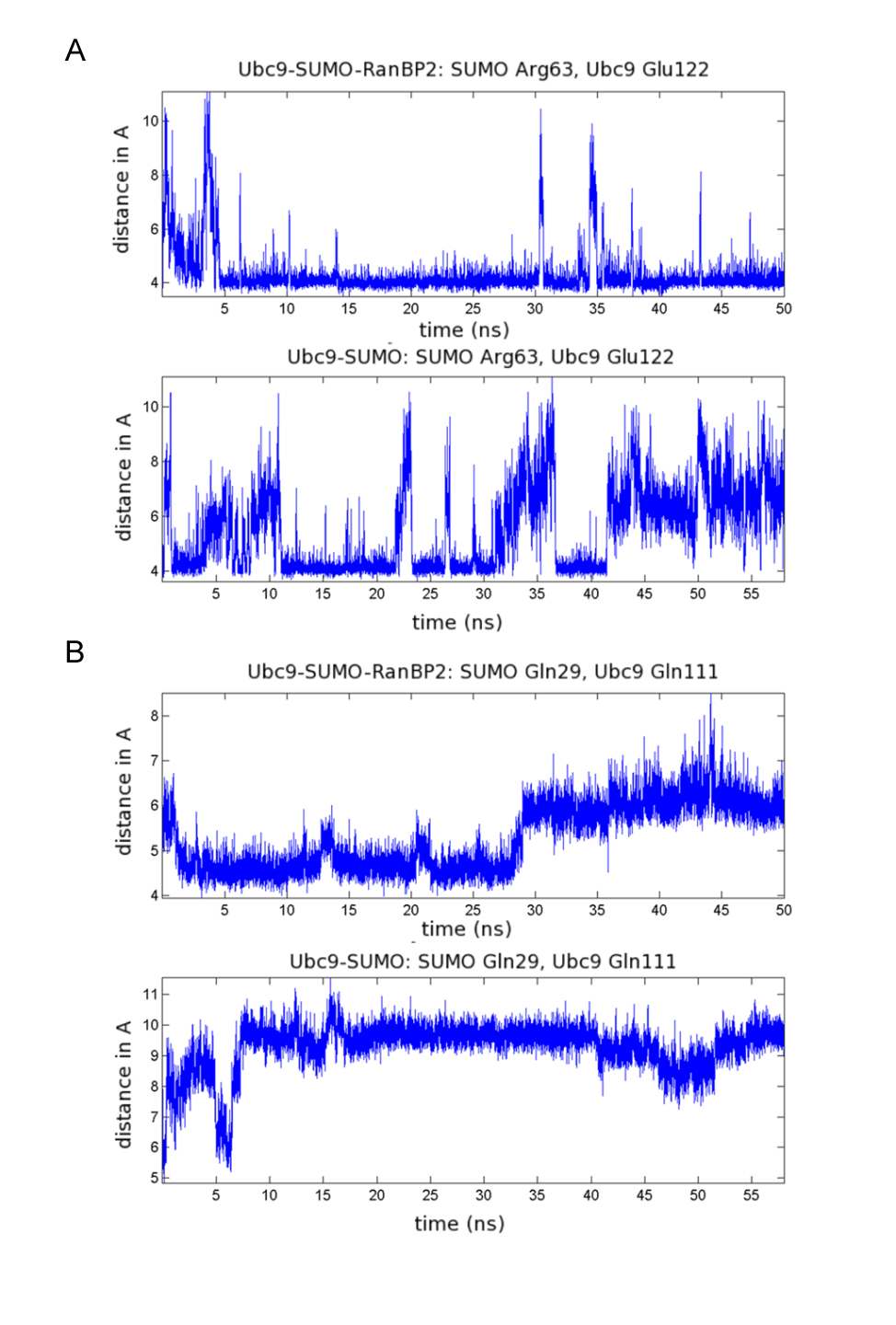

Supplement: Figure S1 — Distances between potential hydrogen bonds. (A) Distance between alpha carbon atoms of residues Arg63 of SUMO and Glu122 of Ubc9 throughout the trajectories. Upper lane is the distance for Ubc9-SUMO-RanBP2 complex, and lower lane is the distance for Ubc9-SUMO complex. (B) Distance between α carbon atoms of residues Gln29 of SUMO and Gln111 of Ubc9 throughout the trajectories. Upper lane is the distance for Ubc9-SUMO-RanBP2 complex, and lower lane is the distance for Ubc9-SUMO complex. (4.23 MB TIF) [file pcbi.1000913.s002.tif]

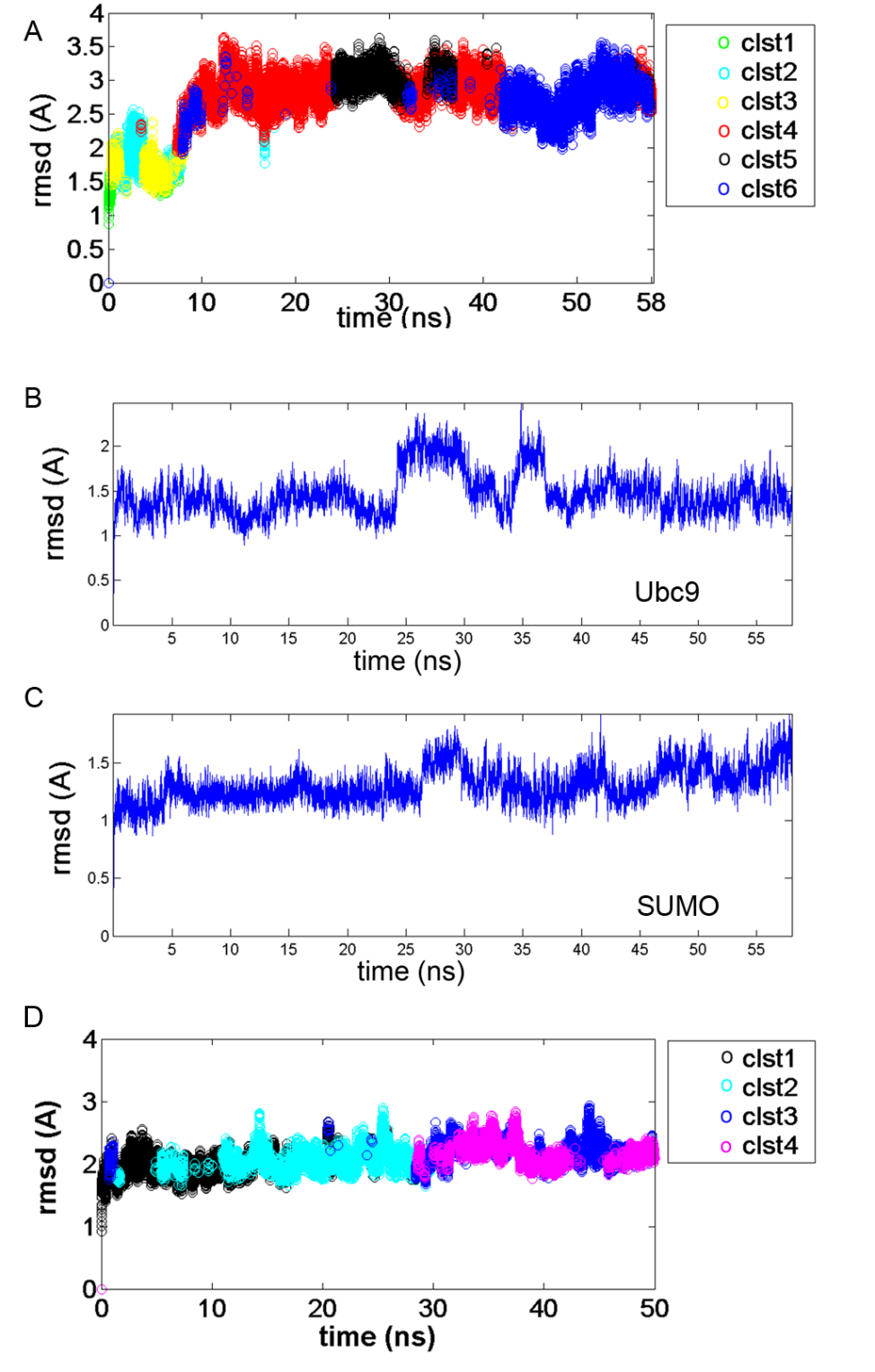

Supplement: Figure S2 — Rmsd values for both complexes throughout the simulation. (A) The rmsd values with the alignment of the whole complex structure, throughout the simulation for Ubc9-SUMO complex. The jump in rmsd can be observed around 10 ns. The rmsd values for the same simulation, calculated by alignment of individual chains are illustrated for (B) Ubc9 and for (C) SUMO. The rmsd jump is not observed in B and C. (D) The rmsd values with the alignment of the whole complex structure, throughout the simulation for Ubc9-SUMO-RanBP2 complex. (4.49 MB TIF) [file pcbi.1000913.s003.tif]

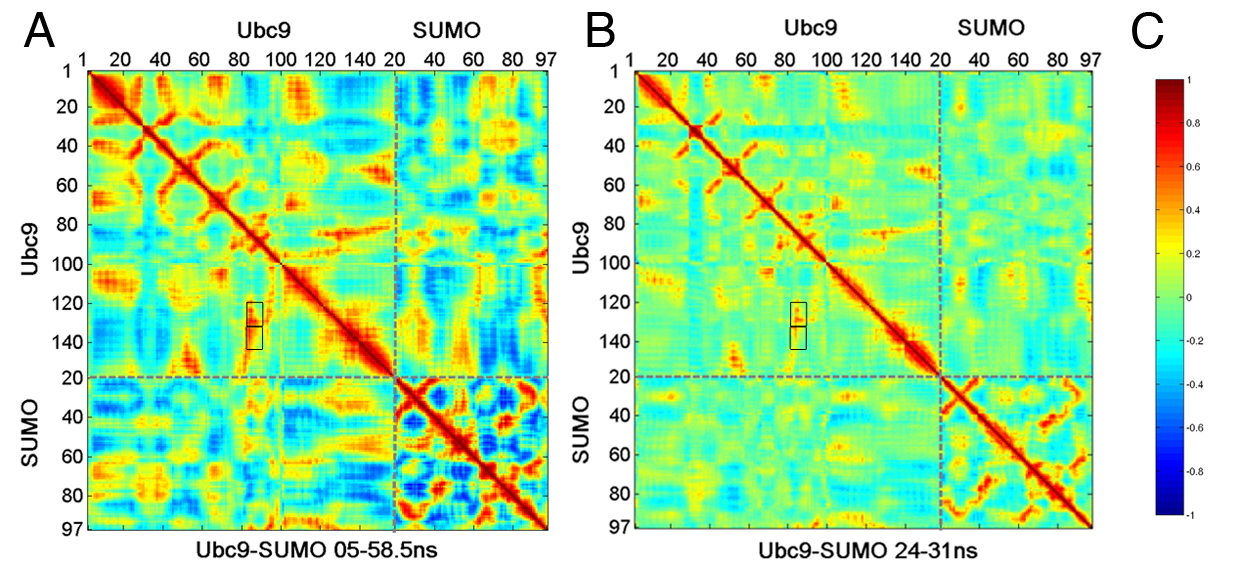

Supplement: Figure S3 — Correlations of mean-square fluctuations. (A) Correlations of Ubc9-SUMO overall trajectory. (B) Correlations of Ubc9-SUMO from Ubc9-SUMO trajectory between 24–31 ns of simulation time. In both A and B, the rectangles surround the correlations between His83-Ser89 and Asn121-Ala131 of Ubc9, and correlations between His83-Ser89 and Ala131-Arg141 of Ubc9. (C) The color bar indicating the correlations for both A and B. (2.14 MB TIF) [file pcbi.1000913.s004.tif]

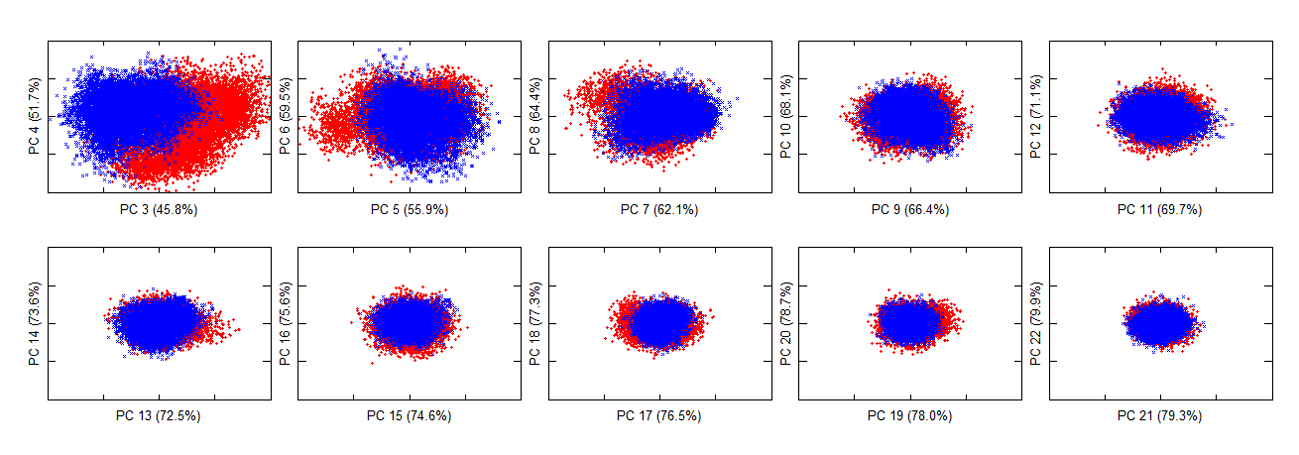

Supplement: Figure S4 — The projections of Ubc9 conformations on principal components. The projections of Ubc9 conformations from Ubc9-SUMO and Ubc9-SUMO-RanBP3 simulations are given in blue and red, respectively. The principal components are given in Å. All plots are in the range [−10∶10] in x- and y-axes. The proportion of all trajectory accounted for accounted for by the PCs up to current PC is given in parenthesis on each axis. (1.80 MB TIF) [file pcbi.1000913.s005.tif]

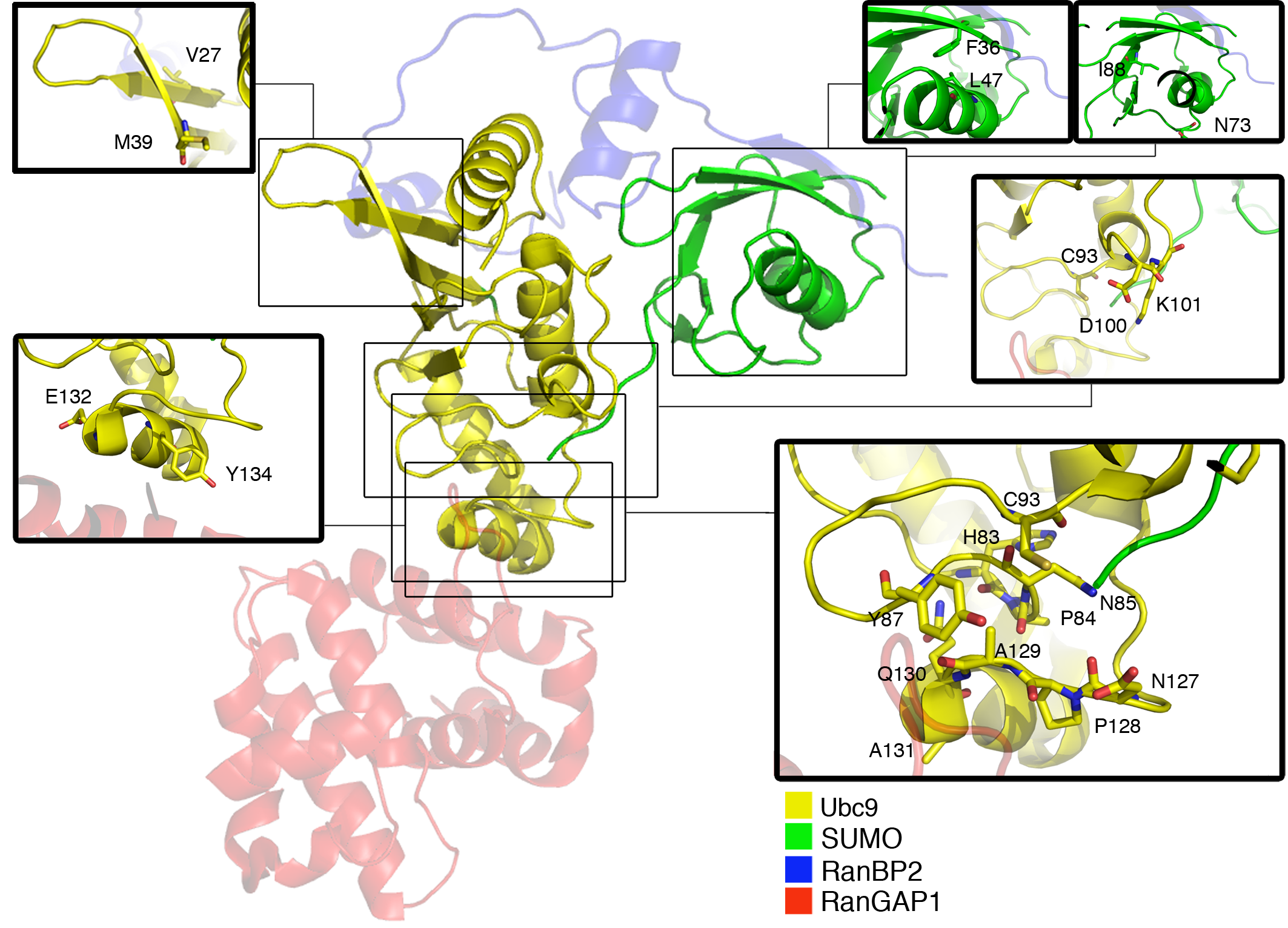

Supplement: Figure S5 — The structure of the Ubc9-SUMO-RanBP2-RanGAP1 complex [20]. This figure is a detailed version of Figure 2 of manuscript. The chains are colored as indicated in the legend. The insets highlight the residue groups that are of interest. Top left: Ubc9 mobile loop Val27 to Glu42. Bottom left: Ubc9 residues Glu132-Arg141, responsible for specific target recognition. Top right: SUMO residues Phe36 to Leu47 and Asp73 to Ile88. These regions mark the proximity of SUMO residues that pack with E3 and the also show correlated fluctuations with Ubc9 residues Val27 to Glu42. Middle right: Ubc9 catalytic Cys93, residues functional in target recognition Asp100, Lys101. Bottom right: Ubc9 HPN (His83-Pro84-Asn85) motif, has a structural role, maintains the hydrogen-bonding networks around the catalytic site of Ubc9. Ubc9 residues which interact with the consensus sumoylation motif, see text for functional details of individual residues. (1.61 MB TIF) [file pcbi.1000913.s006.tif]

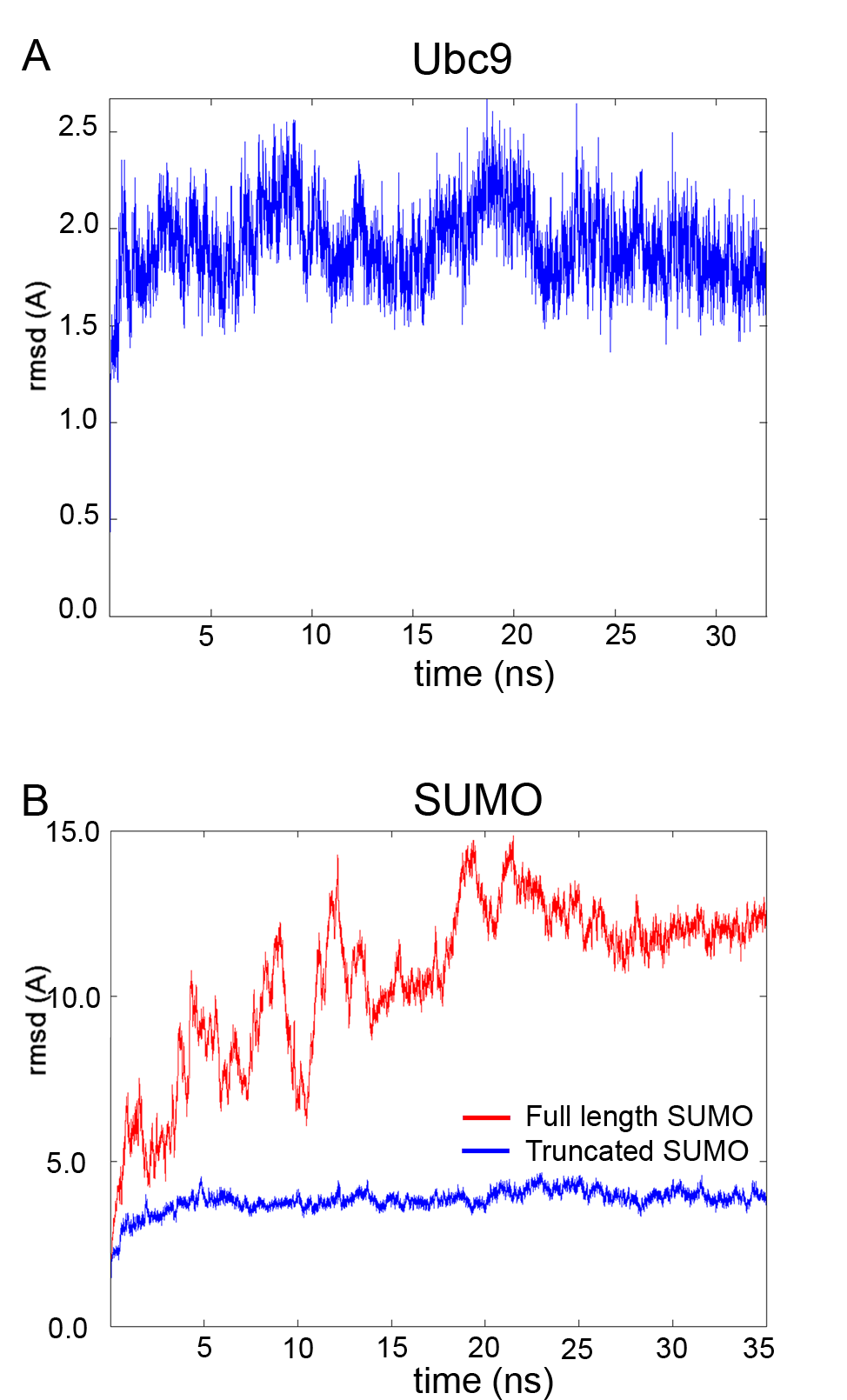

Supplement: Figure S6 — Rmsd values for unbound Ubc9 and SUMO throughout the simulation. (A) The rmsd values of Ubc9. (B) The rmsd values for SUMO. Values for full length protein are displayed in red, values for N-terminal truncated protein are in blue. The effect of the first 21 residues of protein on calculations can be seen from the difference between two plots. The truncated values are used for comparison through text. (0.31 MB TIF) [file pcbi.1000913.s007.tif]
