# Supplementary material for: A Mechanistic View of the Role of E3 in Sumoylation
Source: PLoS Comput Biol. 2010 Aug 26;6(8):e1000913. doi: 10.1371/journal.pcbi.1000913 (PMC2928739; doi:10.1371/journal.pcbi.1000913)
Supplement: Table S1 — RMSD with chain based alignments, second set of simulations. (0.04 MB DOC) [file pcbi.1000913.s008.doc]

Table S1. RMSD with chain based alignments, second set of simulations

| **Ubc9-SUMO-E3 complex** | **Ubc9 rmsd** | **SUMO rmsd** |  |
| --- | --- | --- | --- |
|  | **aligned on Ubc9** | **aligned on SUMO** | **aligned on Ubc9** |
| Representative Structure 1 | 1.18 | 1.01 | 1.75 |
| Representative Structure 2 | 1.19 | 1.21 | 3.08 |
| Representative Structure 3 | 1.15 | 1.25 | 2.25 |
| **Ubc9-SUMO complex** | **Ubc9 rmsd** | **SUMO rmsd** |  |
|  | **aligned on Ubc9** | **aligned on SUMO** | **aligned on Ubc9** |
| Representative Structure 1 | 1.16 | 1.09 | 1.32 |
| Representative Structure 2 | 1.19 | 1.24 | 7.15 |
| Representative Structure 3 | 1.34 | 1.24 | 5.21 |
| Representative Structure 4 | 1.44 | 1.24 | 8.34 |
| Representative Structure 5 | 1.20 | 1.25 | 4.55 |
| Representative Structure 6 | 1.11 | 1.28 | 2.78 |
| Representative Structure 7 | 1.36 | 1.30 | 3.66 |
| Representative Structure 8 | 1.38 | 1.37 | 5.54 |
| Representative Structure 9 | 1.11 | 1.39 | 5.49 |
