# Supplementary material for: A Mechanistic View of the Role of E3 in Sumoylation
Source: PLoS Comput Biol. 2010 Aug 26;6(8):e1000913. doi: 10.1371/journal.pcbi.1000913 (PMC2928739; doi:10.1371/journal.pcbi.1000913)
Supplement: Table S2 — Time windows defined by the clustering analysis. (0.03 MB DOC) [file pcbi.1000913.s009.doc]

Table S2.Time windows defined by the clustering analysis

| Ubc9-SUMO | Ubc9-SUMO-E3 |
| --- | --- |
| Time window (ns) | Time window (ns) |
| 0.5 – 6 | 0.4 – 28 |
| 6 – 12 | 28 – 50 |
| 12 – 24 |  |
| 24 – 31 |  |
| 31 – 42 |  |
| 42 – 58 |  |
